# Chrysophanol Induced Glioma Cells Apoptosis via Activation of Mitochondrial Apoptosis Pathway

**DOI:** 10.1080/21655979.2021.1972079

**Published:** 2021-09-14

**Authors:** Jia Gu, Sunil Rauniyar, Yan Wang, Wenjian Zhan, Chengkun Ye, Shaogan Ji, Guanzheng Liu

**Affiliations:** a Department of Neurosurgery, The Affiliated Hospital of Xuzhou Medical University, China; b Nanjing Medical University, Nanjing, China; c Institute of Nervous System Diseases, Xuzhou Medical University, Xuzhou, China; d The Graduate School, Xuzhou Medical University, Xuzhou, China; e Department of Oncology, The Affiliated Hospital of Xuzhou Medical University, Xuzhou City, China; f Department of Neurosurgery, Kaifeng Central Hospital, Kaifeng City, China

**Keywords:** Chrysophanol, Glioma, Apoptosis, Cell cycle, Mitochondrial Apoptosis Pathway

## Abstract

Glioma is a common intracranial tumor originated from neuroglia cell. Chrysophanol is an anthraquinone derivative proved to exert anticancer effects in various cancers. This paper investigated the effect and mechanism of chrysophanol in glioma. Glioma cell lines U251 and SHG-44 were adopted in the experiments. The cells were treated with chrysophanol at different concentrations (0, 10, 20 50, 100 and 200 μM) for 48 h in the study, and then processed with MitoTempo. Mitochondria and cytosol were isolated to investigate the role of mitochondria during chrysophanol functioning on glioma cells. Cell viability was detected through 3-(4,5-Dimethyl-2-Thiazolyl)-2,5-Diphenyl Tetrazolium Bromide (MTT) assay, and cell apoptosis, cell cycle as well as relative reactive oxygen species (ROS) were assessed by flow cytometry. Expressions of Cytosol Cyt C, cleaved caspase-3, cleaved caspase-9, Cyclin D1 and Cyclin E were evaluated by western blot. In U251 and SHG-44 cells, with chrysophanol concentration rising, cell viability, expressions of Cyclin D1 and Cyclin E were decreased while cell apoptosis, levels of cleaved caspase-3, cleaved caspase-9 and Cytosol Cyt C as well as ROS accumulation were increased with cell cycle arrested in G1 phase. Besides, chrysophanol promoted ROS accumulation, cell apoptosis and transfer of Cyt C from mitochondria to cytosol in cells while MitoTempo partly reversed the effect of chrysophanol. Chrysophanol promoted cell apoptosis via activating mitochondrial apoptosis pathway in glioma.

## Graphical abstract

**Figure:**
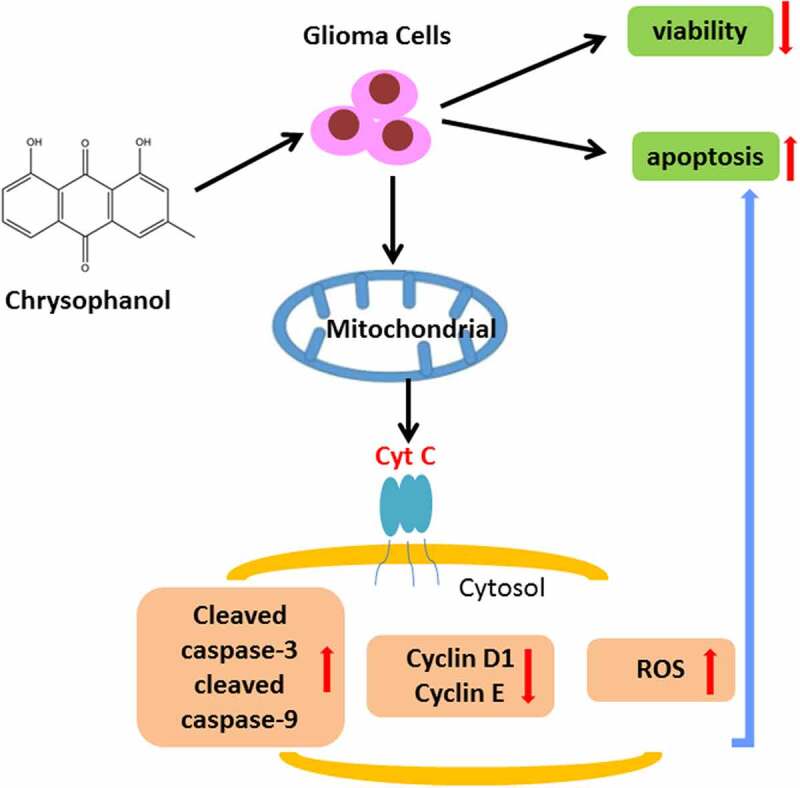

## Introduction

In 2018, nearly 300 thousand people were diagnosed with brain and nervous system tumors and about 240 thousand of them died of the cancer developed from brain and nervous system [1]. Characterized by high proliferation and invasiveness, glioma is the most common primary intracranial tumor [2]. Malignant glioma is the most common type among glioma, accounting for nearly 40%–50% of all brain malignancies [3–5]. Despite advancement has been made in surgery, radiotherapy and chemotherapy in recent years, the frequent recurrence of glioma, poor prognosis and a short survival time pose a great challenge to the treatment of the disease [6–10]. And up to now, as a basis for chemotherapy, Tem-ozolomide (TMZ) has been a conventional agent due to its certain curative efficacy on treating glioma clinically [8,11]. Nevertheless, persistent recurrence and aggressiveness of glioma imply that a single drug cannot solve the malignant tumor completely, let alone the greatly limited efficiency of TMZ due to the drug resistance of glioma cells [8,12,13]. Although recent studies have presented the potential efficacy of several components, such as celastrol for glioma therapy [14], the relative researches in this respect remains little. Hence, it is urgent and necessary to do research for facilitation of drug development and find novel and effective drugs.

Chrysophanol (1, 8-Dihydroxy-3-methyl-9, 10-anthraquinone) is a phytochemical extracted from *Rheum officinale* (rhubarb), which has been utilized as a traditional Chinese herbal medicine. Belonging to the family of anthraquinone (rhein, physcion, aloe-emodin and emodin etc.), chrysophanol is proved to inhibit cell progression [15–20], and has also performed anticancer activity among variety of malignant tumors. For example, chrysophanol promotes cell morphologic changes, induces cell apoptosis through DNA damage and arrests S phase cell cycle in liver cancer [21]. In addition, chrysophanol represses cell proliferation of colon cancer via epidermal growth factor receptor (EGFR)/ mammalian target of rapamycin (mTOR) signaling proteins such as extracellular signal-regulated kinase 1/2 (ERK1/2), P70S6K and AKT [22]. And the similar suppressive function of chrysophanol has been presented on breast, lung and ovarian cancers [15,23–25]. However, the effect and mechanism of chrysophanol on glioma remain unclear.

Apoptosis is a kind of programmed cell death for maintaining homeostasis of internal environment, which is observed during chemotherapy against all types of cancers [26,27]. It is reported that intrinsic pathway, namely mitochondrial pathway, is one of the important signaling cascades controlling cell apoptosis [28,29]. Previous studies have revealed that chrysophanol induces cell apoptosis by mitochondrion-related pathway in lung and ovarian cancer [15,25]. Therefore, we attempted to explore whether chrysophanol exerts an effect on glioma through mitochondrial apoptosis pathway.

In this study, we investigated the role of chrysophanol in glioma at first and then explored the specific mechanism of chrysophanol via targeting mitochondrial pathway, trying to offer a promising therapeutic agent for glioma. Our results demonstrated that chrysophanol promoted cell apoptosis via activating mitochondrial apoptosis pathway in glioma.

## Materials and methods

### Cell culture

Glioma cell lines U251 and SHG-44 were bought from Shanghai Yaji Biological Technology Co., Ltd. (YS301 C; YS266C, Shanghai, China, http://www.yajimall.com/). Cells were cultured in dulbecco’s modified eagle medium (DMEM; PM150210, Procell Life Science&Technology Co., Ltd., Wuhan, China) supplemented with 10% fetal bovine serum (FBS; 164,210, Procell Life Science&Technology Co., Ltd., China) and 1% Benzylpenicillin/Streptomycin (PB180120, Procell Life Science&Technology Co., Ltd., China) in a humidified incubator at 37°C with 5% CO_2_.

### Reagent treatment

In the experiments of effect of chrysophanol on glioma cells, cells were treated with different concentrations of chrysophanol (C_15_H_10_O_4_, ≥98%, 0, 10, 20, 50, 100 and 200 μM; 01542, Sigma-Aldrich, St. Louis, MO, USA) for 48 h. And during the researches on MitoTempo, cells were co-treated with 10 μM chrysophanol and 5 μM MitoTempo (5 μM; SML0737, Sigma-Aldrich, USA) for 24 h.

### 3-(4,5-dimethyl-2-thiazolyl)-2,5-diphenyl tetrazolium bromide (MTT) assay

After trypsin (T1350, Beijing Solarbio Science&Technology Co., Ltd., Beijing, China) digestion, cells were seeded into 96-well plates at a density of 1 × 10^5^ cells/well, and then incubated at 37°C with 5% CO_2_ for 24 h. The culture medium was discarded and the cells were washed two times with PBS. Next, 20 μl MTT solution (5 mg/ml; PB180519, Procell Life Science&Technology Co., Ltd., China) was added into each well to further incubate the cells at 37°C for another 4 h. Subsequently, 150 μl Dimethyl sulfoxide (DMSO; ST1276, Beyotime Biotechnology, Shanghai, China) was added after the removal of MTT solution and plates were put in a shaker at a low speed for 10 min to completely dissolve the crystal (formazan). The absorbance was measured at 570 nm by a microplate reader (HBS-1096 C, NanJing DeTie Laboratory Equipment Co., Ltd., Nanjing, China, http://www.detielab.com/).

### Cell apoptosis assay

Cell apoptosis was detected according to previous research methods [15]. The cell apoptosis was assessed by Annexin V-FITC Apoptosis Detection Kit (CA1020, Beijing Solarbio Science&Technology Co.,Ltd., China). The cells were detached by using trypsin-EDTA, and then collected by centrifugation. After being rinsed with PBS, the cells were then suspended in binding buffer and centrifuged at 300 × g for 10 min. Followed by removal of supernatant, the cells were resuspended in binding buffer at 1 × 10^6^ cells/ml. Annexin V-FITC (5 μL) was added to cell solution and mixed well in a dark room at room temperature for 10 min. And PI (5 μL) was also added into cells under the same conditions for another 5 min. The apoptosis was analyzed through a CytoFLEX flow cytometer (Beckman Coulter, Inc., Brea, CA, USA).

### Cell cycle analysis by flow cytometry

The cell cycle was evaluated with flow cytometry using PI/RNase staining buffer (550,825, BD Biosciences, San Jose, CA, USA). After trypsin digestion, the cells were seeded at a density of 3 × 10^5^ cells per well in 6-well plates. Subsequently, cells were harvested and fixed with 75% ethanol at −20°C overnight. Then PI/RNase staining buffer was added into plates and cells were then incubated at room temperature for another 15 min. The cell cycle was analyzed by BD Accuri™ C6 Plus Flow Cytometer (BD Biosciences, USA) and FlowJo, version 10.7 (BD Biosciences, USA) was applied for data analysis.

### Mitochondria and cytosol isolation assay

The mitochondria and cytosol of cells were separated by mitochondrial extraction kit (SM0020, Beijing Solarbio Science&Technology Co., Ltd., China). Briefly, after being trypsinized, cells were rinsed with PBS and centrifuged at 800 g for 10 min. Then cells (5 × 10^7^) were resuspended in 1 ml ice-precooling lysis buffer, following which the cell suspension was grinded 30 times at 0°C in an ice bath using a glass homogenizer (YA0856, Beijing Solarbio Science&Technology Co., Ltd., China). Then the cell homogenate was centrifuged at 1000 g at 4°C for 5 min and the supernatant was collected. After that, the supernatant was collected again and centrifuged at 12,000 g at 4°C for 10 min with cytosol in the supernatant and mitochondria in the bottom.

### Western blot

Total protein was isolated with cell lysis buffer for Western and IP (P0013, Beyotime Biotechnology, China), cells were centrifuged at 10,000 × g at 4°C for 5 min and the supernatant was collected. Total protein concentration was evaluated by bicinchoninic acid (BCA) assay (P0011, Beyotime Biotechnology, China). Equal contents of protein and ColorMixed Protein Marker (11–180KD) (PR1910, Beijing Solarbio Science&Technology Co., Ltd., Beijing, China) were separated by SDS-polyacrylamide gel electrophoresis (SDS-PAGE) with SDS-PAGE Gel Preparation Kit (P0012AC, Beyotime Biotechnology, China). Subsequently, proteins were transferred onto polyvinylidene fluoride (PVDF) membranes (88,585, Thermo Fisher Scientific, Waltham, MA, USA) and blocked in 5% bovine serum albumin (BSA; PC0001, Beijing Solarbio Science & Technology Co., Ltd., China) for 1 h, and then incubated at 4°C overnight with anti-cleaved caspase-3 (1:500; ab2302, Abcam, Cambridge, MA, USA), anti-cleaved caspase-9 (1:200; ab2324, Abcam, USA), anti-Cyclin D1 (1:200; ab16663, Abcam, USA), anti-Cyclin E (1:1000; ab33911, Abcam, USA), anti-β-actin (1:1000; ab8226, Abcam, USA) and anti-Cyt C (1:5000; ab133504, Abcam, USA) antibodies. After being washed four times with Tris-buffered saline containing Tween 20 (TBST; ST673, Beyotime Biotechnology, China), the membranes were incubated with the corresponding secondary antibodies conjugated to Horseradish Peroxidase (HRP; ab6721, 1:5000; Abcam, USA) at room temperature for 1 h and washed five times with TBST for 5 min. Electrochemiluminescence (ECL) reagent (P0018AS, Beyotime Biotechnology, China) was utilized for protein visualization using the chemiluminescence system (SH-Focus523, Shenhua Bio. Co., Ltd., Hangzhou, China, http://www.shenhuabio.cn/index.php). Image J software, version 1.48 (National Institutes of Health, Bethesda, MD, USA) was employed for western blot analysis.

### Reactive oxygen species (ROS) assay by flow cytometry

ROS levels were measured according to previous research methods [23]. U251 and SHG-44 cells (2 × 10^5^ cells/well) were trypsinized and seeded in 6-well plates. DMSO was used as vehicle control. The cells were collected and then resuspended in 500 ml of 2ʹ, 7ʹ‐dichlorofluorescin diacetate (DCFH-DA; 10 mM; 287,810, Sigma-Aldrich, USA) at 37°C for 20 min to determine ROS. A CytoFLEX flow cytometer was used to measure the fluorescence intensity.

### Statistical analysis

All experiments were repeated independently at least three times. Statistical analysis was detected by Graphpad 8.0 software (GraphPad Software Inc., San Diego, CA, USA). Data were expressed as means ± standard deviation. One-way ANOVA was adopted to compare multiple groups with Tukey’s post hoc test. A statistically significant difference can be accepted when *p* < 0.05.

## Results

### Chrysophanol suppressed cell viability while promoting cell apoptosis in glioma cells

Chrysophanol, an anthraquinone derivative, is proved to exert anti-cancer effects in various cancers. This paper investigated the effect and mechanism of chrysophanol in glioma cells.

**Figure 1.**
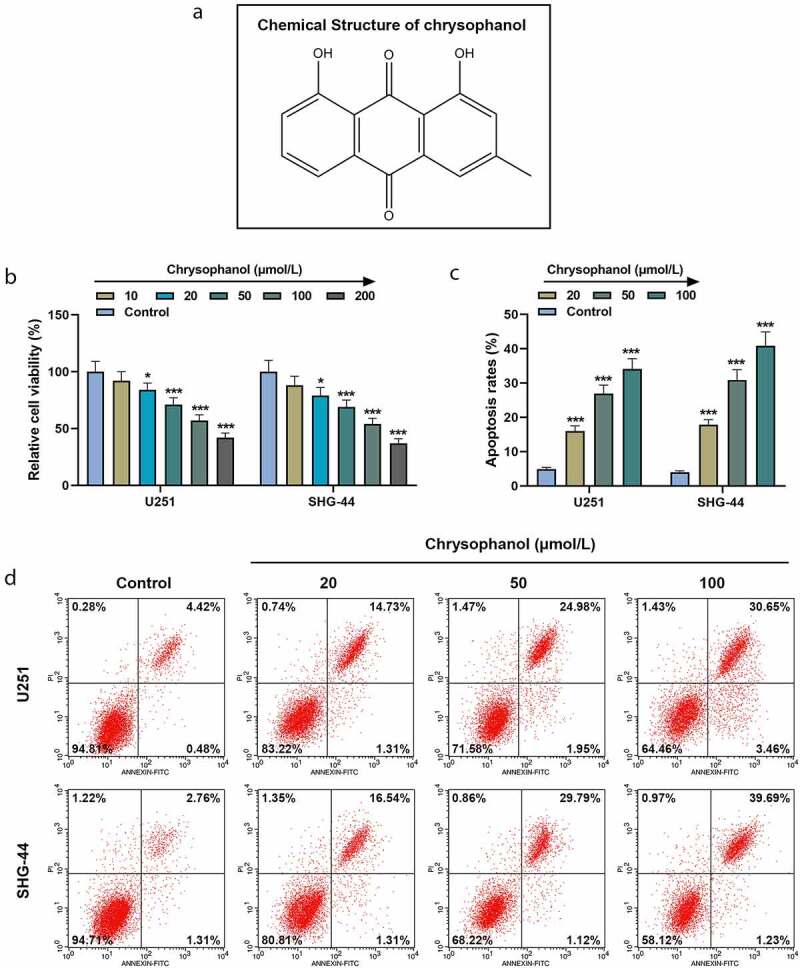
**Chrysophanol suppressed cell viability while promoting cell apoptosis in glioma cells**. (a) The structural formula of chrysophanol. (b) Cell viability at 48 h of U251 and SHG-44 cells was detected by 3-(4,5-Dimethyl-2-Thiazolyl)-2,5-Diphenyl Tetrazolium Bromide (MTT) assay after treatment with different concentration of chrysophanol. (c) Apoptosis rates of U251 and SHG-44 cells were assessed by Annexin V-FITC Apoptosis Detection Kit with flow cytometry after treatment with different concentration of chrysophanol. (d) Representative images of cell apoptosis in U251 and SHG-44 cells through Annexin V-FITC Apoptosis Detection Kit with flow cytometry after treatment with different concentration of chrysophanol. *p < 0.05, *** *p* < 0.001 vs. Control group. All experiments were repeated independently at least three times. Data were expressed as the means ± standard deviation

First, we examined the effects of chrysophanol at different concentrations on glioma cells. As the concentration of chrysophanol rose, cell viabilities of U251 and SHG-44 cells were decreased in comparison with those in control group (Figure 1b, *p* < 0.05). Thereby, we chose 20, 50 and 100 μM as the test concentrations for subsequent experiments. Besides, a marked increase of apoptosis rate in U251 and SHG-44 cells was observed compared with that in control group (Figure 1c and d, *p* < 0.001), suggesting chrysophanol suppressed cell viability and promoted cell apoptosis in a dose-dependent manner.

### Chrysophanol induced cell cycle arrest and increased Cytosol Cyt C expression in glioma cells

**Figure 2.**
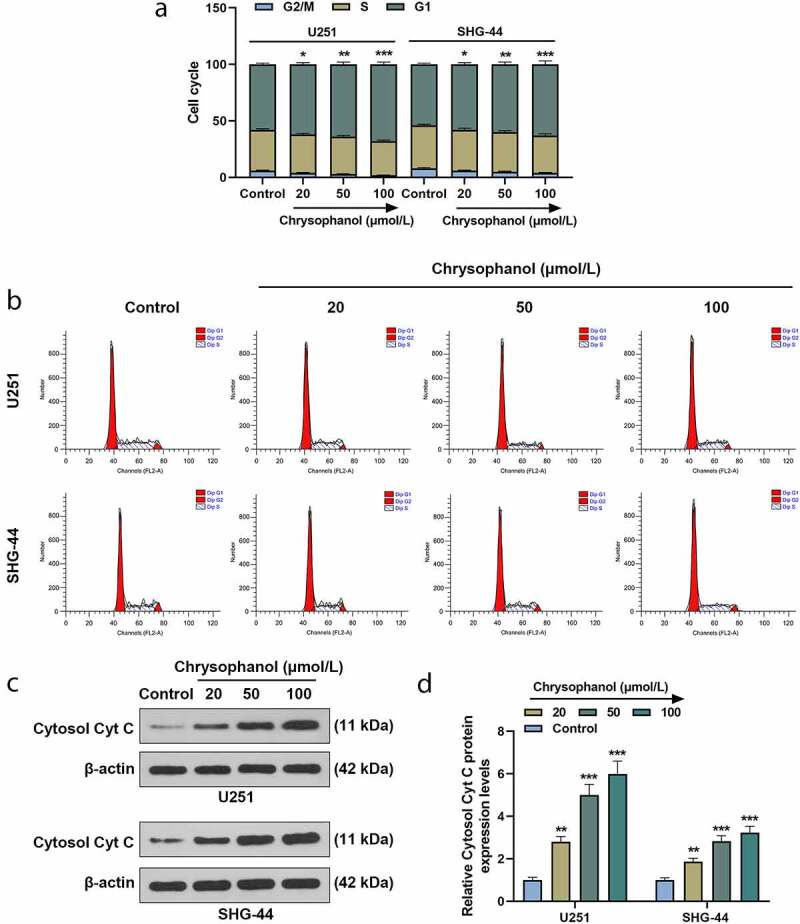
**Chrysophanol induced cell cycle arrest and increased Cytosol Cyt C expression in glioma cells**. (a) Cell cycle of U251 and SHG-44 cells was tested by flow cytometry after treatment with different concentration of chrysophanol. (b) Representative images of cell cycle in U251 and SHG-44 cells using flow cytometry after treatment with different concentration of chrysophanol. (c) Representative images of Cytosol Cyt C protein bands through western blot after treatment with different concentration of chrysophanol. β-actin was used as a loading control. (d) Cytosol Cyt C protein expression levels of U251 and SHG-44 cells were detected by western blot after treatment with different concentration of chrysophanol. β-actin was used as a loading control. *p < 0.05, **p < 0.01, *** *p* < 0.001 vs. Control group. All experiments were repeated independently at least three times. Data were expressed as the means ± standard deviation

Through analysis of flow cytometry, it was found that in comparison with control group, the percentage of G1 phase in U251 and SHG-44 cells treated with chrysophanol was significantly increased in a dose-dependent manner (Figure 2a and b, *p* < 0.05), indicating that chrysophanol arrested cell cycles at G1 phase in glioma cells. Additionally, the protein expression of Cytosol Cyt C in U251 and SHG-44 cells rose with the increase of chrysophanol concentration as compared with that in control group (Figure 2c and d, *p* < 0.01), indicating that chrysophanol promoted the transfer of Cytc C from mitochondria to cytosol.

### Chrysophanol increased cleaved caspase-3 and cleaved caspase-9 expressions as well as ROS accumulation while decreasing Cyclin D1 and Cyclin E levels in glioma cells

**Figure 3.**
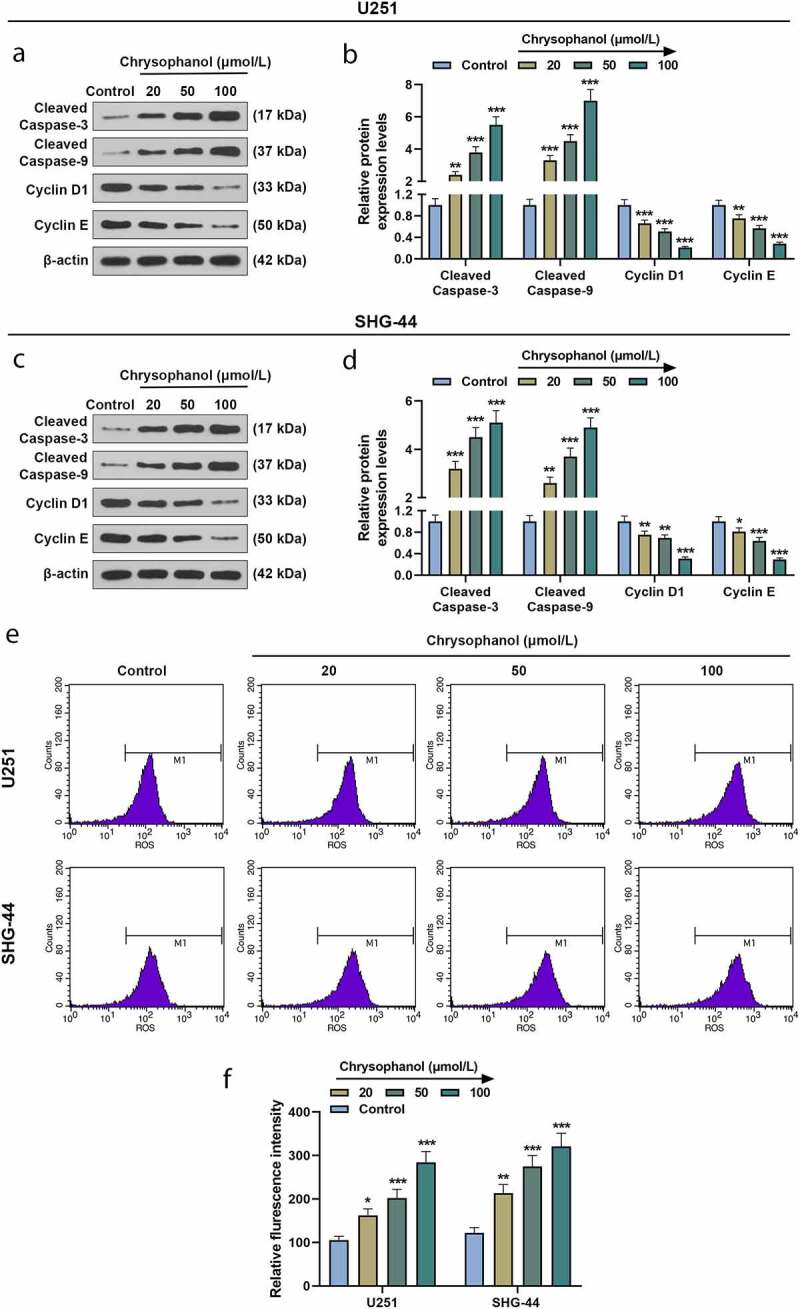
**Chrysophanol increased cleaved caspase-3 and cleaved caspase-9 expressions as well as reactive oxygen species (ROS) accumulation while decreasing Cyclin D1 and Cyclin E levels in glioma cells**. (a and b) Representative images of protein bands (a) as well as protein expression levels of Cleaved caspase-3, cleaved caspase-9, Cyclin D1 and Cyclin E in U251 cells were detected by western blot after treatment with different concentrations of chrysophanol. β-actin was used as a loading control. (c and d) Representative images of protein bands (c) as well as protein expression levels of Cleaved caspase-3, cleaved caspase-9, Cyclin D1 and Cyclin E in SHG-44 cells were detected by western blot after treatment with different concentrations of chrysophanol. β-actin was used as a loading control. (e) Representative images of ROS detection by flow cytometry after treatment with different concentrations of chrysophanol. (f) Flurescence intensity in U251 and SHG-44 was evaluated by flow cytometry after treatment with different concentrations of chrysophanol. * *p* < 0.05, ** *p* < 0.01, *** *p* < 0.001 vs. Control group. All experiments were repeated independently at least three times. Data were expressed as the means ± standard deviation

The results of western blot showed the expressions of apoptosis-related proteins cleaved caspase-3 and cleaved caspase-9 in U251 and SHG-44 cells treated with chrysophanol were higher than those in control group (Figure 3a-d, *p* < 0.05). The expressions of these two apoptosis-related proteins were positively correlated with chrysophanol concentration. In addition, cell cycle-related proteins Cyclin D1 and Cyclin E levels were evidently reduced in U251 and SHG-44 cells treated with chrysophanol when compared with those in control group (Figure 3a-d, *p* < 0.05). The chrysophanol concentration-dependent reduced Cyclin D1 and Cyclin E expressions. Furthermore, chrysophanol at all tested concentrations led to an obvious increase of flurescence intensity in U251 and SHG-44 cells during ROS assay by flow cytometry compared with control group (Figure 3e and f, *p* < 0.05), and the intensity of flurescene rose with the increase of chrysophanol concentration, indicating that chrysophanol promoted ROS accumulation of mitochondria in glioma cells.

### MitoTempo partly reversed the effect of chrysophanol on promoting ROS accumulation of mitochondria and inducing cell apoptosis in glioma cells

**Figure 4.**
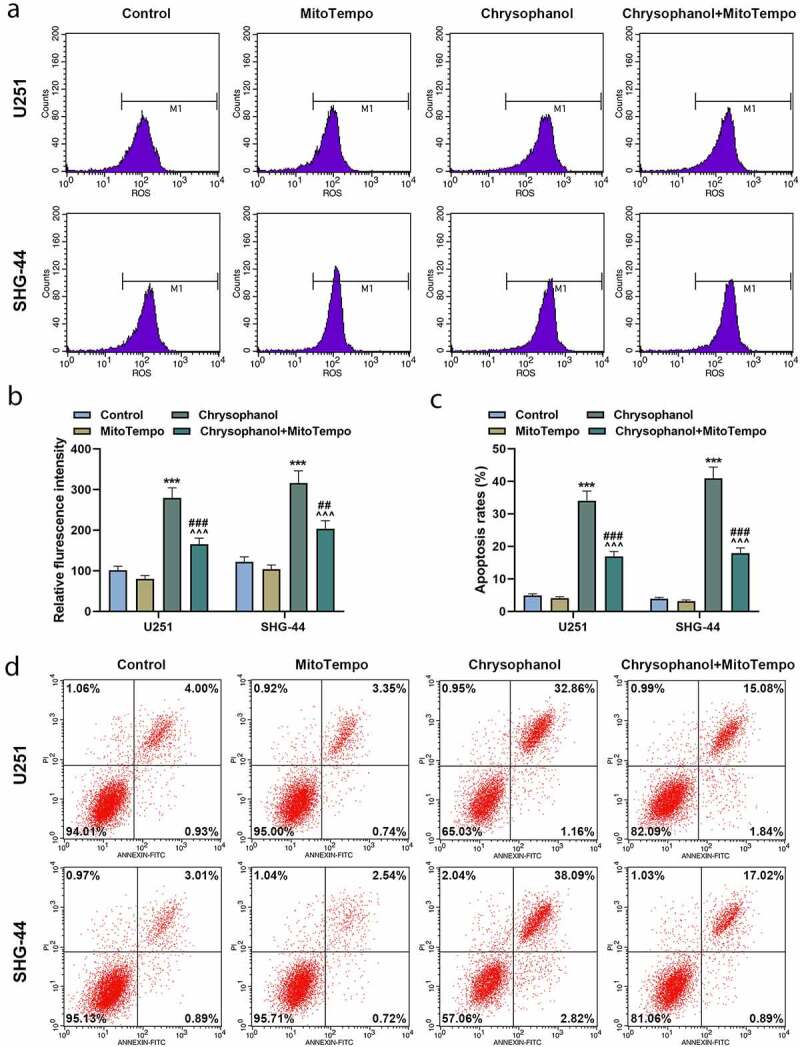
**MitoTempo partly reversed the effect of chrysophanol on promoting reactive oxygen species (ROS) accumulation of mitochondria and inducing cell apoptosis in glioma cells**. (a) Representative images of ROS detection by flow cytometry after treatment with chrysophanol and MitoTempo. (b) Flurescence intensity in U251 and SHG-44 was evaluated by flow cytometry after treatment with chrysophanol and MitoTempo. (c) Apoptosis rates of U251 and SHG-44 cells were assessed by Annexin V-FITC Apoptosis Detection Kit with flow cytometry after treatment with chrysophanol and MitoTempo. (d) Representative images of cell apoptosis in U251 and SHG-44 cells through Annexin V-FITC Apoptosis Detection Kit with flow cytometry after treatment with chrysophanol and MitoTempo. *** *p* < 0.001 vs. Control group; ^^^^^ *p* < 0.001 vs. Chrysophanol group; ## *p* < 0.01, ^###^ *p* < 0.001 vs. MitoTempo group. All experiments were repeated independently at least three times. Data were expressed as the means ± standard deviation

The fluorescence intensity of U251 and SHG-44 cells in Chrysophanol+ MitoTempo group was lower than that in chrysophanol group but was higher than that in MitoTempo group (Figure 4a and b, *p* < 0.01). Meanwhile, intensity of fluorescence was increased in chrysophanol group in comparison with that in control group (Figure 4a and b, *p* < 0.01), which suggested that MitoTempo partly offset the effect of chrysophanol on promoting ROS accumulation of mitochondria in glioma cells. Moreover, the apoptosis rates of U251 and SHG-44 cells in chrysophanol group were greatly increased compared with those in control and Chrysophanol+MitoTempo group (Figure 4c and d, *p* < 0.001), while a prominent rising of cell apoptosis in Chrysophanol+MitoTempo group was observed in comparison with that in MitoTempo group (Figure 4c and d, *p* < 0.001).

### MitoTempo partly reversed the effect of chrysophanol on increaing leakage of Cytc C from mitochondria to cytosol in glioma cells

**Figure 5.**
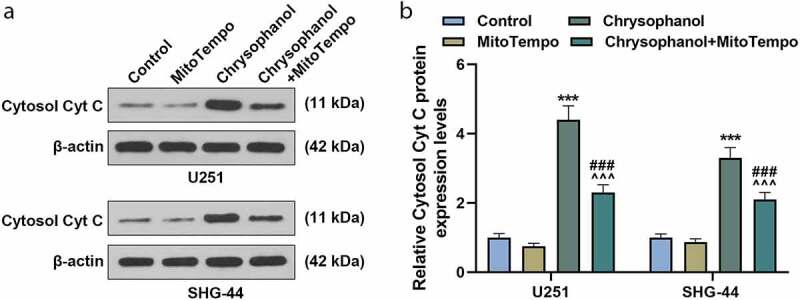
**MitoTempo partly reversed the effect of chrysophanol on advancing leakage of Cytc C from mitochondria to cytosol in glioma cells**. (a) Representative images of Cytosol Cyt C protein bands through western blot after treatment with chrysophanol and MitoTempo. β-actin was used as a loading control. (b) Cytosol Cyt C protein expression levels of U251 and SHG-44 cells were detected by western blot after treatment with chrysophanol and MitoTempo. β-actin was used as a loading control. *** *p* < 0.001 vs. Control group; ^^^^^ *p* < 0.001 vs. Chrysophanol group; ^###^ *p* < 0.001 vs. MitoTempo group. All experiments were repeated independently at least three times. Data were expressed as the means ± standard deviation

U251 and SHG-44 cells showed a higher protein expression of Cytosol Cyt C when treated with chrysophanol and MitoTempo than the cells treated with MitoTempo (Figure 5a and b, *p* < 0.001), while the cells treated with chrysophanol presented an increased protein level of Cytosol Cyt C in comparsion with those in control and Chrysophanol+ MitoTempo groups (Figure 5a and b, *p* < 0.001), indicating that MitoTempo could partly reverse the function of chrysophanol on facilitating leakage of Cytc C from mitochondria to cytosol.

## Discussion

Glioma is a kind of brain tumor in human central nervous system with high mortality and relapse rates [3,30]. And more evidence has been accumulated to validate the repressive function of chrysophanol on cell development [15,21–25]. For example, it has been reported that chrysophanol inhibits the osteoglycin/mTOR and activates NF2 signaling pathways to reduce viability and proliferation of malignant meningioma cells [31]. Nevertheless, so far the effect and mechanism of chrysophanol on glioma have remained unclear. As an active process of cell death, apoptosis can be triggered through mitochondrial apoptosis pathway [27,28]. What’s more, it has been reported that chrysophanol has anticancer activities, including induction of cell apoptosis by mitochondrial apoptosis pathway [15,25]. Thereby, we presumed that chrysophanol might play a part in glioma cells via mitochondrial pathway. And this study elucidated the role and mechanism of chrysophanol on glioma.

Firstly, we probed into the role of chrysophanol on glioma cells. In consistent with the researches about the function of chrysophanol on breast cancer cells [24], chrysophanol significantly reduced the cell viability while increasing cell apoptosis in a dose-dependent manner, indicating the protective effect of chrysophanol on glioma. Additionally, the results of cell cycle assay showed that pretreatment with chrysophanol was connected with a marked rise of the cell number in G1 phase, suggesting that chrysophanol arrested cell cycle in G1 phase.

It has been proved that the release of Cyt C from mitochondria to cytosol induces cell apoptosis [32]. And as a group of primary drivers for cell apoptosis, caspases are synthesized as inactive zymogens within the cell until they were cleaved with apoptotic signaling stimuli. Besides, former studies have revealed that the release of Cyt C activates caspase-9 and further causes the activation of caspase-3, which is associated with mitochondria pathway [33–37]. The cell cycle-related proteins Cyclin D1 and Cyclin E, which regulate cell cycle during the transition from G1 to S phase, are involved in cell apoptosis among development of many malignancies [38–44]. During the experiments, we found that the protein expressions of Cytosol Cyt C, cleaved caspase-3 and cleaved caspase-9 were obviously increased, whereas those of Cyclin D1 and Cyclin E were declined dramatically. Therefore, chrysophanol might induce cell apoptosis through up-regulating Cytosol Cyt C, cleaved caspase-3 and cleaved caspase-9 expressions while down-regulating Cyclin D1 and Cyclin E expressions.

There are two major pathways during cell apoptosis including the extrinsic (death receptor) and intrinsic (mitochondrial) pathways [45,46]. The intrinsic apoptosis pathway modulates the permeability of mitochondrial outer membrane and can produce pores in the membrane, which leads to the release of apoptosis-related factors like Cyt C [47]. ROS is a vital molecule directly implicated in regulating mitochondrial functions, the accumulation of which has been reported to induce cell apoptosis through various mechanisms, such as cellular injury and cell cycle arrest induced by oxidative DNA damage [48–52]. Besides, another paper reported that chrysophanol-induced cell apoptosis by promoting ROS generation through mitochondrial apoptosis pathway in choriocarcinoma, and anticancer drugs also increased ROS levels, leading to cell death in glioma cells [53,54]. In line with the results above, our study discovered that chrysophanol increased ROS accumulation in mitochondria of glioma cells, implicating that chrysophanolmight induced ROS accumulation to promote release of Cyt C from mitochondria to cytoplasm, which caused apoptosis of glioma cells.

Secondly, MitoTempo, an antioxidant targeting mitochondria, was applied in this part as a reversing verification to determine whether the mitochondrial apoptosis pathway participated in the process of chrysophanol functioning on glioma cells. The results showed MitoTempo partly reversed the effect of chrysophanol on accumulating ROS of mitochondria, inducing cell apoptosis and promoting leakage of Cyt C from mitochondria to cytosol in glioma, validating the positive role of chrysophanol against glioma by mitochondrial apoptosis pathway. In addition, it has been reported that chrysophanol inhibits EMT formation and metastasis via a Wnt-3-dependent signaling pathway in oral squamous cell carcinoma [55]. Chrysophanol selectively represses breast cancer cell growth by inducing reactive oxygen species production and endoplasmic reticulum stress via AKT and mitogen-activated protein kinase signal pathways [23]. Chrysophanol inhibits proliferation and induces apoptosis through NF-κB/cyclin D1 and NF-κB/Bcl-2 signaling cascade in breast cancer cell lines [24]. In choriocarcinoma, chrysophanol induces apoptosis through regulation of ROS and the AKT and ERK1/2 pathways [54]. Chrysophanol may play an anti-cancer role in cancer by regulating multiple signaling pathways.

All in all, the observational data acquired in the research demonstrated the protective effect of chrysophanol on glioma and revealed its mechanism, offering powerful support for function of chrysophanol on malignant tumors. Besides, this study provided a possible feasibility of utilizing promising mitochondrial apoptosis pathway as a therapeutic target and adopting chrysophanol as a possible agent for interference and treatment of glioma. In the future, we will test the effect of chrysophanol on normal cells and explore the function of chrysophanol *in vivo* to validate the conclusions of this study. We will also continue to search for more anti-tumor signaling pathways of chrysophanol to provide more possible treatments for the treatment of glioma.

## Limitation

The roles of chrysophanol in normal cells and in animals were not assessed in our study, leaving the safety of chrysophanol in doubt. Moreover, the comparison on clinical efficacy of chrysophanol and conventional chemotherapy needs further study to improve the management of glioma.

## Conclusion

In summary, the study showed that chrysophanol could induce cell apoptosis and promote cell cycle arrest to suppress the development of glioma. And further researches revealed that the chrysophanol regulated the anticancer effect on glioma cells via mitochondrial apoptosis pathway activation. The results of the paper strengthened the understanding on activities of chrysophanol, indicating that chrysophanol may serve as an innovative chemotherapeutic agent for glioma.

## Supplementary Material

Supplemental Material

## Data Availability

The analyzed data sets generated during the study are available from the corresponding author on reasonable request.

## References

[1] Bray F, Ferlay J, Soerjomataram I, et al. Global cancer statistics 2018: GLOBOCAN estimates of incidence and mortality worldwide for 36 cancers in 185 countries. CA Cancer J Clin. 2018;68:394–424. 30207593 10.3322/caac.21492

[2] Schwartzbaum JA, Fisher JL, Aldape KD, et al. Epidemiology and molecular pathology of glioma. Nat Clin Pract Neurol. 2006;2(9):494–503. quiz 1 p following 16. 16932614 10.1038/ncpneuro0289

[3] Dunn GP, Rinne ML, Wykosky J, et al. Emerging insights into the molecular and cellular basis of glioblastoma. Genes Dev. 2012;26:756–784. 22508724 10.1101/gad.187922.112 PMC3337451

[4] Louis DN, Perry A, Reifenberger G, et al. The 2016 world health organization classification of tumors of the central nervous system: a summary. Acta Neuropathol. 2016;131:803–820. 27157931 10.1007/s00401-016-1545-1

[5] Omuro A, DeAngelis LM. Glioblastoma and other malignant gliomas: a clinical review. Jama. 2013;310:1842–1850. 24193082 10.1001/jama.2013.280319

[6] Cloughesy TF, Cavenee WK, Mischel PS, et al. Glioblastoma: from molecular pathology to targeted treatment. Annu Rev Pathol. 2014;9:1–25. 23937436 10.1146/annurev-pathol-011110-130324

[7] Ostrom QT, Gittleman H, Stetson L, et al. Epidemiology of gliomas. Cancer Treat Res. 2015;163:1–14. 25468222 10.1007/978-3-319-12048-5_1

[8] Stavrovskaya AA, Shushanov SS, Rybalkina EY, et al. Problems of glioblastoma multiforme drug resistance. Biochem Biokhimiia. 2016;81:91–100. 10.1134/S0006297916020036 27260389

[9] Wen PY, Kesari S. Malignant gliomas in adults. N Engl J Med. 2008;359(5):492–507. 18669428 10.1056/NEJMra0708126

[10] Xiong J, Zhou LI, Lim Y, et al. Mature brain-derived neurotrophic factor and its receptor TrkB are upregulated in human glioma tissues. Oncol Lett. 2015;10:223–227. 26171003 10.3892/ol.2015.3181 PMC4487187

[11] Siegel RL, Miller KD, Jemal A, et al. Cancer statistics, 2017. CA Cancer J Clin. 2017;67:7–30. 28055103 10.3322/caac.21387

[12] Altieri R, Fontanella M, Agnoletti A, et al. Role of Nitric Oxide in glioblastoma therapy: another step to resolve the terrible puzzle?. Transl Med UniSa. 2015;12:54–59. 26535188 PMC4592044

[13] Liao A, Shi R, Jiang Y, et al. SDF-1/CXCR4 axis regulates cell cycle progression and epithelial-mesenchymal transition via up-regulation of survivin in glioblastoma. Mol Neurobiol. 2016;53:210–215. 25421212 10.1007/s12035-014-9006-0

[14] Liu X, Zhao P, Wang X, et al. Celastrol mediates autophagy and apoptosis via the ROS/JNK and Akt/mTOR signaling pathways in glioma cells. J Exp Clin Cancer Res. 2019;38(1):184. 31053160 10.1186/s13046-019-1173-4 PMC6500040

[15] Lim W, An Y, Yang C, et al. Chrysophanol induces cell death and inhibits invasiveness via mitochondrial calcium overload in ovarian cancer cells. J Cell Biochem. 2018;119(12):10216–10227. 30129050 10.1002/jcb.27363

[16] Lu J, Xu Y, Zhao Z, et al. Emodin suppresses proliferation, migration and invasion in ovarian cancer cells by down regulating ILK in vitro and in vivo. Onco Targets Ther. 2017;10:3579–3589. 28790850 10.2147/OTT.S138217 PMC5530856

[17] Wang J, Liu S, Yin Y, et al. FOXO3-mediated up-regulation of bim contributes to rhein-induced cancer cell apoptosis. Apoptosis. 2015;20(3):399–409. 25501496 10.1007/s10495-014-1071-3

[18] Wang Y, Luo Q, He X, et al. Emodin induces apoptosis of colon cancer cells via induction of autophagy in a ROS-dependent manner. Oncol Res. 2018;26:889–899. 28762328 10.3727/096504017X15009419625178 PMC7844792

[19] Wu YY, Zhang JH, Gao JH, et al. Aloe-emodin (AE) nanoparticles suppresses proliferation and induces apoptosis in human lung squamous carcinoma via ROS generation in vitro and in vivo. Biochem Biophys Res Commun. 2017;490:601–607. 28629998 10.1016/j.bbrc.2017.06.084

[20] Xie MJ, Ma YH, Miao L, et al. Emodin-provoked oxidative stress induces apoptosis in human colon cancer HCT116 cells through a p53-mitochondrial apoptotic pathway. Asian Pac J Cancer Prev. 2014;15:5201–5205. 25040975 10.7314/apjcp.2014.15.13.5201

[21] Ni CH, Chen PY, Lu HF, et al. Chrysophanol-induced necrotic-like cell death through an impaired mitochondrial ATP synthesis in Hep3B human liver cancer cells. Arch Pharm Res. 2012;35:887–895. 22644856 10.1007/s12272-012-0514-z

[22] Lee MS, Cha EY, Sul JY, et al. Chrysophanic acid blocks proliferation of colon cancer cells by inhibiting EGFR/mTOR pathway. Phytother Res. 2011;25(6):833–837. 21089180 10.1002/ptr.3323

[23] Park S, Lim W, Song G, et al. Chrysophanol selectively represses breast cancer cell growth by inducing reactive oxygen species production and endoplasmic reticulum stress via AKT and mitogen-activated protein kinase signal pathways. Toxicol Appl Pharmacol. 2018;360:201–211. 30300626 10.1016/j.taap.2018.10.010

[24] Ren L, Li Z, Dai C, et al. Chrysophanol inhibits proliferation and induces apoptosis through NF-κB/cyclin D1 and NF-κB/Bcl-2 signaling cascade in breast cancer cell lines. Mol Med Rep. 2018;17:4376–4382. 29344652 10.3892/mmr.2018.8443 PMC5802211

[25] Zhang J, Wang Q, Wang Q, et al. Chrysophanol exhibits anti-cancer activities in lung cancer cell through regulating ROS/HIF-1a/VEGF signaling pathway. Naunyn-Schmiedeberg’s Arch Pharmacol. 2020;393:469–480. 31655854 10.1007/s00210-019-01746-8

[26] Byrne GI, Ojcius DM. Chlamydia and apoptosis: life and death decisions of an intracellular pathogen. Nature Rev Microbiol. 2004;2(10):802–808. 15378044 10.1038/nrmicro1007

[27] Zimmermann KC, Bonzon C, Green DR, et al. The machinery of programmed cell death. Pharmacol Ther. 2001;92(1):57–70. 11750036 10.1016/s0163-7258(01)00159-0

[28] Lee JY, Park JY, Kim DH, et al. Erigeron annuus protects PC12 neuronal cells from oxidative stress induced by ROS-mediated apoptosis. Evid Based Complement Alternat Med. 2020;2020:3945194. 31998396 10.1155/2020/3945194 PMC6970001

[29] Li X, Song H, Kong F, et al. Pemetrexed exerts anticancer effects by inducing G(0)/G(1)-phase cell cycle arrest and activating the NOXA/Mcl-1 axis in human esophageal squamous cell carcinoma cells. Oncol Lett. 2019;17:1851–1858. 30675247 10.3892/ol.2018.9753 PMC6341790

[30] Ohtaki S, Wanibuchi M, Kataoka-Sasaki Y, et al. ACTC1 as an invasion and prognosis marker in glioma. J Neurosurg. 2017;126:467–475. 27081897 10.3171/2016.1.JNS152075

[31] Wang J, Lv P. Chrysophanol inhibits the osteoglycin/mTOR and activats NF2 signaling pathways to reduce viability and proliferation of malignant meningioma cells. Bioengineered. 2021;12(1):755–762. 33622177 10.1080/21655979.2021.1885864 PMC8291820

[32] Hüttemann M, Pecina P, Rainbolt M, et al. The multiple functions of cytochrome c and their regulation in life and death decisions of the mammalian cell: from respiration to apoptosis. Mitochondrion. 2011;11(3):369–381. 21296189 10.1016/j.mito.2011.01.010 PMC3075374

[33] Ly JD, Grubb DR, Lawen A, et al. The mitochondrial membrane potential (deltapsi(m)) in apoptosis; an update. Apoptosis. 2003;8(2):115–128. 12766472 10.1023/a:1022945107762

[34] Parrish AB, Freel CD, Kornbluth S, et al. Cellular mechanisms controlling caspase activation and function. Cold Spring Harb Perspect Biol. 2013;5(6):a008672. 10.1101/cshperspect.a008672 PMC3660825 23732469

[35] Kothakota S, Azuma T, Reinhard C, et al. Caspase-3-generated fragment of gelsolin: effector of morphological change in apoptosis. Science (New York, NY). 1997;278:294–298. 10.1126/science.278.5336.294 9323209

[36] Launay S, Hermine O, Fontenay M, et al. Vital functions for lethal caspases. Oncogene. 2005;24(33):5137–5148. 16079910 10.1038/sj.onc.1208524

[37] Suzuki N, Urano J, Tamanoi F, et al. Farnesyltransferase inhibitors induce cytochrome c release and caspase 3 activation preferentially in transformed cells. Proc Natl Acad Sci U S A; 1998; 95(26), 15356–15361. 10.1073/pnas.95.26.15356 PMC28047 9860973

[38] Brumby AM, Zraly CB, Horsfield JA, et al. Drosophila cyclin E interacts with components of the brahma complex. EMBO J. 2002;21:3377–3389. 12093739 10.1093/emboj/cdf334 PMC126084

[39] Castro RE, Amaral JD, Solá S, et al. Differential regulation of cyclin D1 and cell death by bile acids in primary rat hepatocytes. Am J Physiol Gastrointest Liver Physiol. 2007;293:G327–34. 17431217 10.1152/ajpgi.00093.2007

[40] El-Kady A, Sun Y, Li YX, et al. Cyclin D1 inhibits whereas c-Myc enhances the cytotoxicity of cisplatin in mouse pancreatic cancer cells via regulation of several members of the NF-κB and Bcl-2 families. J Carcinog. 2011;10:24. 22190866 10.4103/1477-3163.90437 PMC3243339

[41] Hou X, Liang RB, Wei JC, et al. Cyclin D1 expression predicts postoperative distant metastasis and survival in resectable esophageal squamous cell carcinoma. Oncotarget. 2016;7:31088–31096. 27145270 10.18632/oncotarget.9078 PMC5058741

[42] Li Z, Wang C, Prendergast GC, et al. Cyclin D1 functions in cell migration. Cell Cycle (Georgetown, Tex). 2006;5(21):2440–2442. 10.4161/cc.5.21.3428 17106256

[43] Park GH, Song HM, Jeong JB, et al. The coffee diterpene kahweol suppresses the cell proliferation by inducing cyclin D1 proteasomal degradation via ERK1/2, JNK and GKS3β-dependent threonine-286 phosphorylation in human colorectal cancer cells. Food Chem Toxicol. 2016;95:142–148. 27424123 10.1016/j.fct.2016.07.008

[44] Yuan C, Zhu X, Han Y, et al. Elevated HOXA1 expression correlates with accelerated tumor cell proliferation and poor prognosis in gastric cancer partly via cyclin D1. J Exp Clin Cancer Res. 2016;35(1):15. 26791264 10.1186/s13046-016-0294-2 PMC4721151

[45] Ichim G, Tait SW. A fate worse than death: apoptosis as an oncogenic process. Nat Rev Cancer. 2016;16:539–548. 27364482 10.1038/nrc.2016.58

[46] Mace PD, Riedl SJ, Salvesen GS, et al. Caspase enzymology and activation mechanisms. Methods Enzymol. 2014;544:161–178. 24974290 10.1016/B978-0-12-417158-9.00007-8

[47] Cheng MH, Pan CY, Chen NF, et al. Piscidin-1 induces apoptosis via mitochondrial reactive oxygen species-regulated mitochondrial dysfunction in human osteosarcoma cells. Sci Rep. 2020;10:5045. 32193508 10.1038/s41598-020-61876-5 PMC7081333

[48] Ray PD, Huang BW, Tsuji Y, et al. Reactive oxygen species (ROS) homeostasis and redox regulation in cellular signaling. Cell Signal. 2012;24:981–990. 22286106 10.1016/j.cellsig.2012.01.008 PMC3454471

[49] Chen HM, Chang FR, Hsieh YC, et al. A novel synthetic protoapigenone analogue, WYC02-9, induces DNA damage and apoptosis in DU145 prostate cancer cells through generation of reactive oxygen species. Free Radic Biol Med. 2011;50:1151–1162. 21256211 10.1016/j.freeradbiomed.2011.01.015

[50] Lv L, Zheng L, Dong D, et al. Dioscin, a natural steroid saponin, induces apoptosis and DNA damage through reactive oxygen species: a potential new drug for treatment of glioblastoma multiforme. Food Chem Toxicol. 2013;59:657–669. 23871826 10.1016/j.fct.2013.07.012

[51] Morel I, Lescoat G, Cillard J, et al. Kinetic evaluation of free malondialdehyde and enzyme leakage as indices of iron damage in rat hepatocyte cultures. Involvement of free radicals. Biochem pharmacol. 1990;39(11):1647–1655. 2344365 10.1016/0006-2952(90)90107-v

[52] Wang J, Yi J. Cancer cell killing via ROS: to increase or decrease, that is the question. Cancer Biol Ther. 2008;7(12):1875–1884. 18981733 10.4161/cbt.7.12.7067

[53] Rinaldi M, Caffo M, Minutoli L, et al. ROS and brain gliomas: an overview of potential and innovative therapeutic strategies. Int J Mol Sci. 2016;17(6):984. 10.3390/ijms17060984 PMC4926513 27338365

[54] Lim W, Yang C, Bazer FW, et al. Chrysophanol induces apoptosis of choriocarcinoma through regulation of ROS and the AKT and ERK1/2 pathways. J Cell Physiol. 2017;232(2):331–339. 27171670 10.1002/jcp.25423

[55] Chung PC, Hsieh PC, Lan CC, et al. Role of chrysophanol in epithelial-mesenchymal transition in oral cancer cell lines via a Wnt-3-dependent pathway. Evid Based Complement Alternat Med. 2020;2020:8373715. 33014112 10.1155/2020/8373715 PMC7512067

