# Supplementary figures and images for: Chrysophanol Induced Glioma Cells Apoptosis via Activation of Mitochondrial Apoptosis Pathway
Source: Bioengineered. 2021 Sep 14;12(1):6855–68. doi: 10.1080/21655979.2021.1972079 (PMC8806913; doi:10.1080/21655979.2021.1972079)

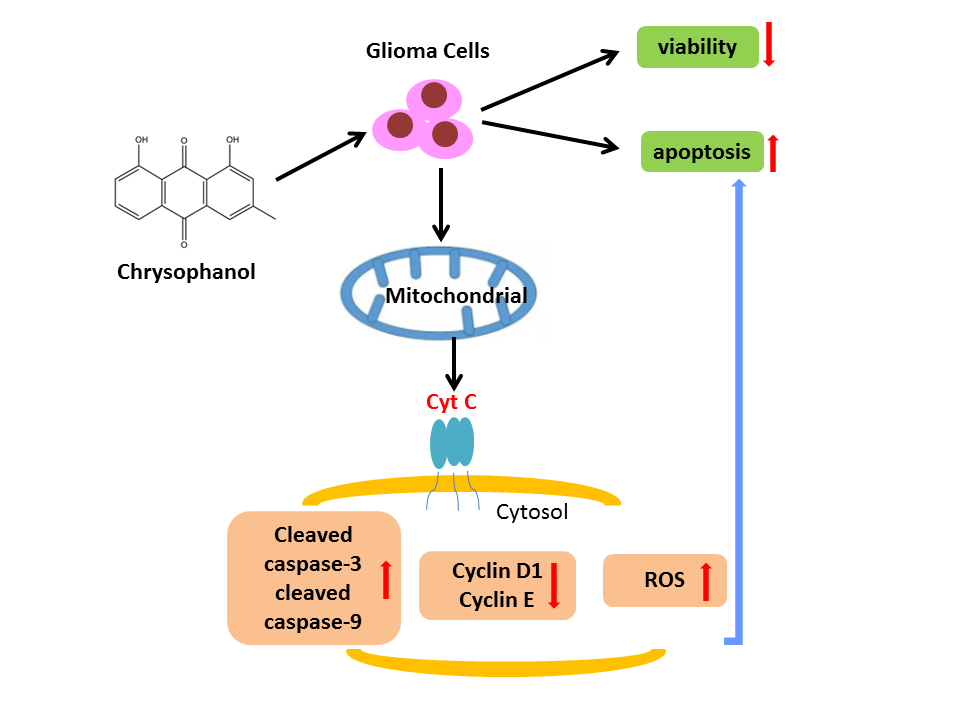

Supplement: Supplemental Material [file KBIE_A_1972079_SM2806.tif]
